# Fab-Arm Exchange Combined with Selective Protein A Purification Results in a Platform for Rapid Preparation of Monovalent Bispecific Antibodies Directly from Culture Media

**DOI:** 10.3390/pharmaceutics12010003

**Published:** 2019-12-18

**Authors:** James Steinhardt, Yanli Wu, Ryan Fleming, Ben T. Ruddle, Pooja Patel, Herren Wu, Changshou Gao, Nazzareno Dimasi

**Affiliations:** Antibody Discovery and Protein Engineering, AstraZeneca, One MedImmune Way, Gaithersburg, MD 201878, USA; james.steinhardt@astrazeneca.com (J.S.); yanli.wu2018@gmail.com (Y.W.); ryan.fleming@astrazeneca.com (R.F.); ben.ruddle@astrazeneca.com (B.T.R.); pooja.patel@astrazeneca.com (P.P.); herren.wu@astrazeneca.com (H.W.); changshou.gao@astrazeneca.com (C.G.)

**Keywords:** monovalent bispecific antibodies, Fab-arm exchange, protein A binding, high-throughput bispecific generation

## Abstract

Bispecific antibody (bsAb) applications have exponentially expanded with the advent of molecular engineering strategies that have addressed many of the initial challenges, including improper light chain pairing, heterodimer purity, aggregation, and pharmacokinetics. However, the lack of high-throughput methods for the generation of monovalent bsAbs has resulted in a bottleneck that has hampered their therapeutic evaluation, as current technologies can be cost-prohibitive and impractical. To address this issue, we incorporated single-matched point mutations in the CH3 domain to recapitulate the physiological process of human IgG4 Fab-arm exchange to generate monovalent bsAbs. Furthermore, we utilized the substitutions H435R and Y436F in the CH3 domain of IgG1, which incorporates residues from human IgG3, thus ablating protein A binding. By exploiting this combination of mutations and optimizing the reduction and reoxidation conditions for Fab arm exchange, highly pure monovalent bsAbs can be rapidly purified directly from combined culture media using standard protein A purification. This methodology, reported herein for the first time, allows for the high-throughput generation of monovalent bsAbs, thus increasing the capacity for evaluating monovalent bsAb iterations for therapeutic potential.

## 1. Introduction

Monoclonal antibodies (mAbs) are homodimeric globular proteins containing two identical light chains and two heavy chains. mAbs are derived from a single B-cell clone and are bivalent molecules whose paratope, which is primarily determined by the variable regions, recognizes the same epitope. Initially, hybridoma technology provided a convenient and simple platform for the generation of monoclonal antibodies [1]. Additional technologies, such as Epstein–Barr virus (EBV) immortalization, phage display, transgenic mice, and single B-cell cloning, have since been utilized to isolate monoclonal antibodies against virtually any given target [2,3,4,5]. The first Food and Drug Administration (FDA) approved monoclonal antibody was OKT3, a mouse IgG2a anti-human CD3 antibody, which was employed as a transplant rejection drug in 1986 [6]. Currently, over five hundred mAbs are at various clinical phases, with over sixty in late-stage clinical studies [7]. Over eighty mAbs have been granted marked approval by the FDA and European Medicinal Agency (EMA) for a multitude of therapeutic indications [8].

Due to the complexity of many human diseases, the dual targeting capacity of engineered bispecific antibodies (bsAbs) significantly expands the therapeutic potential of antibody-based regimens [9,10]. For the treatment of cancer, bsAbs have a potential advantage over mAbs due to their exquisite specificity, which may allow for the specific targeting of discrete tumor populations as well as simultaneous modulation of multiple signaling pathways necessary for aberrant cell growth and survival [11]. Furthermore, the genetic diversity of many pathogenic viruses has significantly limited the therapeutic efficacy of mAbs, which can potentially be overcome by targeting multiple distinct epitopes with bsAbs [12,13,14]. Finally, bsAbs, have shown great potential for immune-modulation through the recruitment of effector cells to clear aberrant cells [9,11]. Two bispecific antibodies are approved for clinical use: Blinatumomab and Emicizumab [15,16,17]. Blinatumomab, a CD19XCD3 bispecifc T-cell engager (BiTE), is approved for patients with relapsed or refractory acute lymphoblastic leukemia. Emicizumab, which by cross-linking factors IX and X restores the coagulation factor VIII, is approved for the treatment of hemophilia A.

Considering the strong rationale for bsAbs, much effort has been made to generate bsAbs in both monovalent and bivalent formats. The first generation of bsAbs were formed using hybrid hybridomas (quadromas) and chemical cross-linking, but these technologies suffered from both a manufacturing and clinical efficacy standpoint [18,19,20,21,22]. More recent efforts have focused on the recombinant expression of bsAbs in various formats [23]. Many of these formats utilize amino acids linkers to generate bivalent bsAbs, such as mAb-domain antibodies (dAb) [24]. Additional formats, such as diabodies, which completely lack a Fragment crystallizable (Fc) region, have been applied to Bispecific T-cell engager (BiTE) purposes [25] and have even been developed in a manner that allows for them to be rapidly screened without any need for purification [26]. In addition to antibody engineering approaches, a variety of bispecific antibodies have been prepared using chemical engineering approaches [27].

However, while these non-traditional formats addressed some of the issues observed with the first generation of bsAbs and are not amenable to high-throughput screening, there is still a demand for rapid preparation platforms for the development of monovalent bsAbs, as they typically retain mAb-like properties including the long in vivo half-life and the ability to elicit Fc-effector functions. The first monovalent bsAbs were generated using knob-into-hole technology in the CH3 region of the Fc to promote heterodimerization [28]. One of the main limitations of this technology was improper light chain pairing, which was later remedied with CrossMAb technology, whereby the CH1 region and CL1 region of one arm are swapped [29]. Additional technologies have since been developed for the generation of monovalent bsAbs with properly paired light chains [30], including tethered-variable CLBsIgG (tcBsIgG) technology, which utilizes a (G_4_S)_4_ linker between the VL and VH [31], and iMab, an IgG1 domain-tethering approach to guide the correct pairing of 2 light and 2 heavy chains, derived from 2 different antibodies [32]. However, most of these bsAb technologies have structural limitations that prevent their use for high-throughput screening purposes [23].

Here, we describe a method for the rapid generation of monovalent bsAbs directly from culture media by combining a single-matched point mutation in the CH3 domain to promote heterodimerization via controlled Fab-arm exchange (cFAE) [33], and by incorporating the H435R and Y436F mutations in the CH3 domain to ablate protein A binding in one arm of the heterodimer to facilitate bsAb purification [34]. Using this approach, highly pure monovalent bsAbs can be rapidly generated and purified directly from combined culture media using standard protein A purification.

## 2. Materials and Methods

### 2.1. General

Size-exclusion chromatography (SEC), reversed-phase liquid chromatography (RPLC), and liquid chromatography mass spectrometry (LCMS) were carried out with an Agilent 1290 Infinity II high pressure liquid chromatography HPLC system equipped with an autosampler and diode array detector (Agilent, Santa Clara, CA, USA). ChemStation software (Agilent, Santa Clara, CA, USA) was used to control the analytics systems and analyze the data. For both SEC and LCMS, an absorbance of 280 nm was used to detect antibodies, while for RPLC, an absorbance of 214 nm was used. Antibodies were quantified using a Nanodrop (Thermo Fischer Scientific, Waltham, MA, USA), using an absorbance of 280 nm and an extinction coefficient of 1.6.

### 2.2. Transient Expression of Parental Antibodies

The following antibodies were used: negative control antibody (NIP228, AstraZeneca), anti-human epidermal growth factor receptor antibody Trastuzumab (HER2), anti-epidermal growth factor receptor antibody Panitumumab (EGFR)**,** and the anti-insulin-like growth factor receptor type 1 antibody (IGF1R, AstraZeneca). Two different variations of each antibody were cloned to contain either the F405L or K409R mutation to promote cFAE [33]. Furthermore, antibodies containing the K409R mutation also contained the H435R and Y436F mutations to ablate protein A binding in one arm of the heterodimer [34]. Antibodies were cloned into a proprietary AstraZeneca mammalian expression vector [35], and transient expression was carried out using suspension-adapted chinese hamster ovary (CHO) cells [36]. Expression was carried out for 14 days using an AstraZeneca proprietary culture medium. The expression level in the culture supernatant was determined using a protein A binding method [35,37]. After expression, the cells were pelleted, discarded after centrifugation, and the culture medium was filtered with 0.2-micron polyethersulfone Rapid-FlowTM 75mm Filter Units (Thermo Scientific, Waltham, MA, USA).

### 2.3. Generation of the Bispecific Antibodies (bsAb)

The concentration of parental antibodies in culture media was quantitated via Biolayer Light Interferometry (BLI) using ForteBio anti-human Fab-CH1 second Generation (FAB2G) biosensors on an Octet RED96 instrument (ForteBio; Pall Life Sciences, Fremont, CA, USA). Then, cultures from F405L and K409R RF constructs were mixed at a ratio of 1:1.2 and then reduced using 75 mM of β-mercaptoethanol (βME) for 5 h at 31 °C before dialysis in phosphate-buffered saline(PBS) at 4 °C overnight with Slide-A-Lyzer™ Dialysis Flasks, 10K molecular weight cut-off (MWCO), 250 mL (Thermo Scientific™, Waltham, MA, USA). Antibodies were then affinity purified using 5mL MabSelect™ SuRe™ (General Electric Healthcare, Chicago, IL, USA) using the manufacturer’s specifications. After elution, bsAbs were filtered using a 0.22 μm syringe filter (Pall Corporation, New York, NY, USA).

### 2.4. Analytical Characterization of the bsAb

Analytical SEC of bsAbs was carried out by using 100 μg (100 μL volume) of antibodies to identify the monomeric content as well as the aggregate and fragment levels. Analytes were loaded into a TSKgel G3000WXL column (Tosoh Biosciences). SEC analysis was performed using 0.1 M sodium sulfate, 0.1 M sodium phosphate, and 10% isopropanol, with pH 6.8. The flow rate was 1 mL/min, and each analysis was carried out for 20 min at room temperature. An absorbance of 280 nm was used during SEC. Intact and reduced reverse phase chromatography (RP-HPLC) was used to estimate the heterodimer efficiency and heterodimer composition by integrating the eluted peaks at 214 nm. bsAbs were reduced at room temperature using 50 mM dithiothreitol (DTT) in PBS pH 7.2. Reduced bsAbs (30 μg) were loaded onto a polymeric reverse-phase media (PLRP-S), 1000 Å column (2.1 × 50 mm, Agilent), and eluted at 80 °C at a flow rate of 1 mL/min with a gradient of 5 to 100% mobile phase B for 25 min (mobile phase A, 0.1% trifluoroacetic acid in water; mobile phase B, 0.1% trifluoroacetic acid in acetonitrile). In addition to RP-HPLC, intact and reduced LCMS was used to estimate the heterodimer efficiency and heterodimer composition. Prior to intact LCMS, monovalent bsAbs were digested using Endo S (Genovisis) for 1 h at 37 °C [38]. LCMS was performed on an Agilent 1290 series ultra-high-performance liquid chromatography (UHPLC) coupled to an Agilent 6230 time of flight (TOF). Five micrograms of intact or reduced antibodies were loaded onto a Zorbax RRHD 300-Diphenyl (2.1 × 50mm, 1.8 μm, Agilent) and eluted at a flow rate of 0.5 mL/min using a step gradient of 80% B after 2.1 min (mobile phase A, 0.1% formic acid in water; mobile phase B, 0.1% formic acid in acetonitrile). A positive time-of-flight mass spectrometry MS scan was acquired, and data collection and processing were carried out using MassHunter software (Agilent).

### 2.5. Cuncurrent Binding of the bsAb

Biolayer light interferometry (BLI) was performed using an Octet RED96 instrument (ForteBio; Pall Life Sciences). Bispecific binding was confirmed by first capturing biotin labeled HER2 or EGFR (R&D System) (ligand 1) at 10 µg/mL onto Streptavidin biosensors for 180 s. The biosensors were then submerged in binding buffer (PBS/0.2% polyethylene glycol sorbitan monolaurate (TWEEN 20) for a wash for 60 s followed by immersion in a solution containing 500 nM of either the parental or bsAbs for 120 s, followed by another wash and subsequent immersion in a solution containing 30 μg/mL of either EGFR or IGF1R (R&D System) (ligand 2) for 120 s for each respective dual binding assay. Biotinylation of probes was performed using EZ-Link NHS-Biotin (ThermoFisher) following the manufacturer’s specifications.

## 3. Results

**Figure 1:**
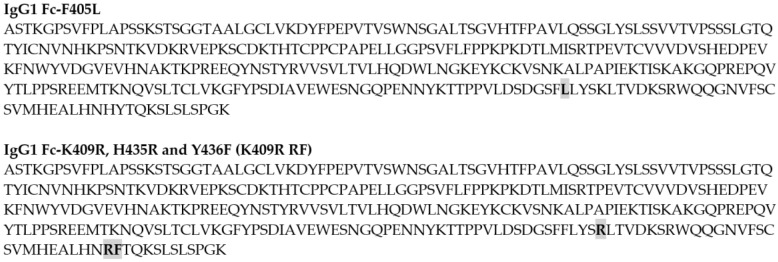
Amino acid sequences of the IgG1 Fc-F405L and IgG1 Fc-K409R, H435R, and Y436F (K409R RF), with the respective mutations bolded and highlighted in grey.

**Figure 2:**
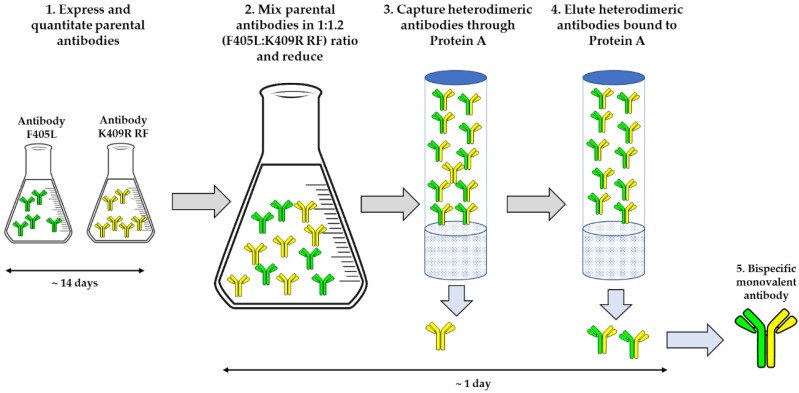
Schematic of the process and timing for generating monovalent bispecific antibodies (bsAbs) directly from cell culture supernatants.

To generate monovalent bsAbs, parental antibodies were first cloned so that their Fc regions contained either the F405L or the K409R, H435R, and Y436F (K409R RF) mutations (Figure 1). These parental antibodies were then expressed, and culture supernatants were quantified via OCTET using anti-human Fab-CH1 second Generation (FAB2G) biosensors on an Octet RED96 instrument. After quantification, parental F405L and K409R RF antibody supernatants were mixed at a ratio of 1:1.2 and reduced using 75 mM of β-mercaptoethanol (βME) at 31 °C for 5 h. The mixture was then dialyzed in PBS overnight at 4 °C. The resulting monovalent bsAb in supernatant was then purified using Protein A MabSelect™ SuRe™, thus allowing excess non-heterodimerized K409R RF antibodies, which are unable to bind Protein A resins due to the incorporation of the H435R and Y436F mutations, to be removed from the final product (Figure 2).

**Figure 3:**
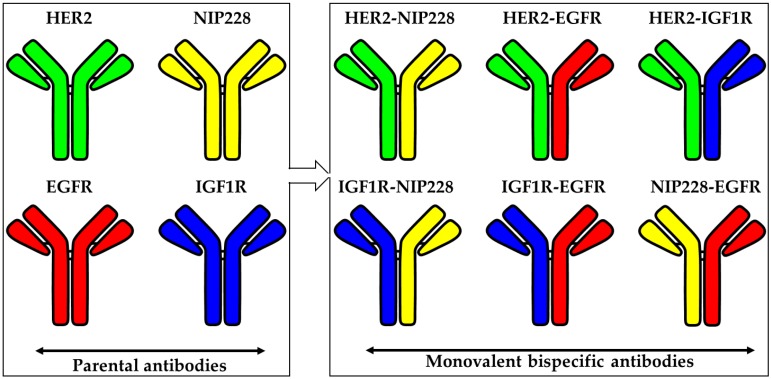
Diagram of monovalent bsAbs combinations described herein that can be obtained from the parental mAbs.

To demonstrate proof-of-concept, we chose four parental antibodies to generate six unique monovalent bsAbs. Here, we selected NIP228 antibody as a negative control, anti-HER2 reactive antibody Trastuzumab (HER2), the epidermal growth factor receptor (EGFR) reactive antibody Panitumumab (Panix), and the IGFR1 reactive antibody TZ1A. Of these antibodies, Trastuzumab was selected to consistently harbor the F405L mutation, as it is a VH3 antibody that would bind to Protein A resins, even if it incorporated the complementing K409R RF mutations. The panel of bsAbs generated were: HER2 F405L-NIP228 K409R RF, HER2 F405L-Panix K409R RF, HER2 F405L-TZ1A K409R RF, TZ1A F405L-NIP228 K409R RF, TZ1A F405L-Panix K409R RF, and NIP228 F405L-Panix K409R RF (Figure 3).

**Figure 4:**
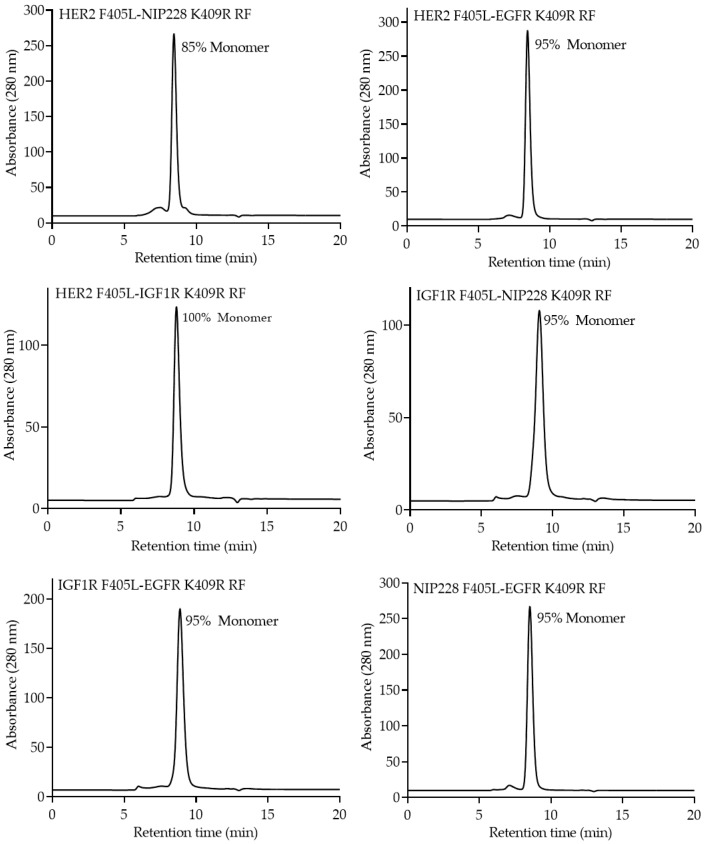
Size-exclusion chromatography (SEC) profiles of the purified monovalent bsAbs. Percentage of monomeric content for each bsAb is shown.

After following the procedure described above to transiently express and purify monovalent bsAbs with Protein A, antibodies were characterized for monomer content via size-exclusion chromatography (SEC) (Figure 4). Overall, purified monovalent bsAbs had monomer contents of over 95% except for Her2 F405L_NIP228 K409R RF, which had higher than expected aggregate content (Figure 4, Top Left Panel). Additionally, all constructs had negligible fragments (Figure 4).

**Figure 5:**
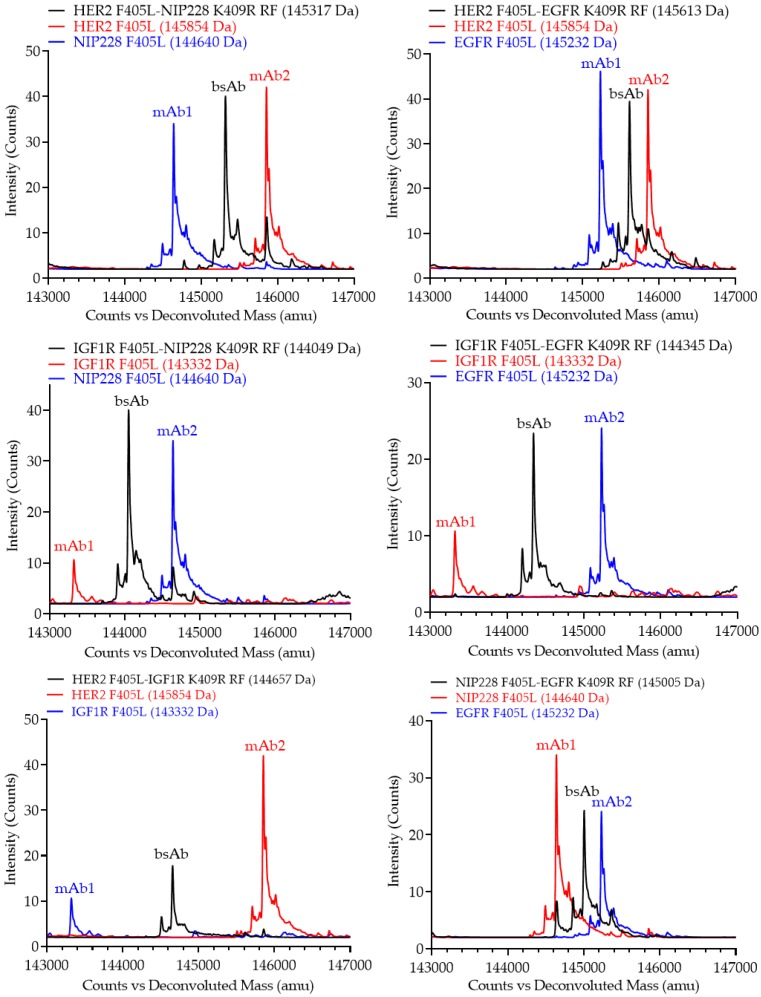
Intact mass spectrometry (MS) profiles of purified monovalent bsAbs (black traces) compared to the two parental mAbs (red and blue trace). All the antibodies were pretreated with Endo S. Molecular weight in Dalton of the parental antibodies and bsAb are shown.

Monovalent bsAbs were digested using Endo S, an endoglycosidase for specifically cleaving the N-linked glycans from the chitobiose core of the heavy chain [37], and then analyzed via intact liquid chromatography-mass spectrometry (LCMS). As Endo S doesn’t not completely remove glycosylation, all antibodies still contained one Fucose and N-Acetylglucosamine on both Fcs, thereby increasing the observed molecular weight by 656 to 692 da depending on the presence of water entities, which are roughly 18 da. Intact LCMS confirmed the predicted molecular weight of the heterodimerized monovalent bsAb species (black trace, Figure 5) as compared to the two parental antibodies (red and blue trace, Figure 5) for all six monovalent bsAbs, as the intact molecular weights of heterodimerized monovalent bsAb species fall exactly between the two parental molecular weights. Furthermore, there are no other identifiable peaks above background for any of the heterodimerized monovalent bsAb species, confirming high heterodimer purity.

**Figure 6:**
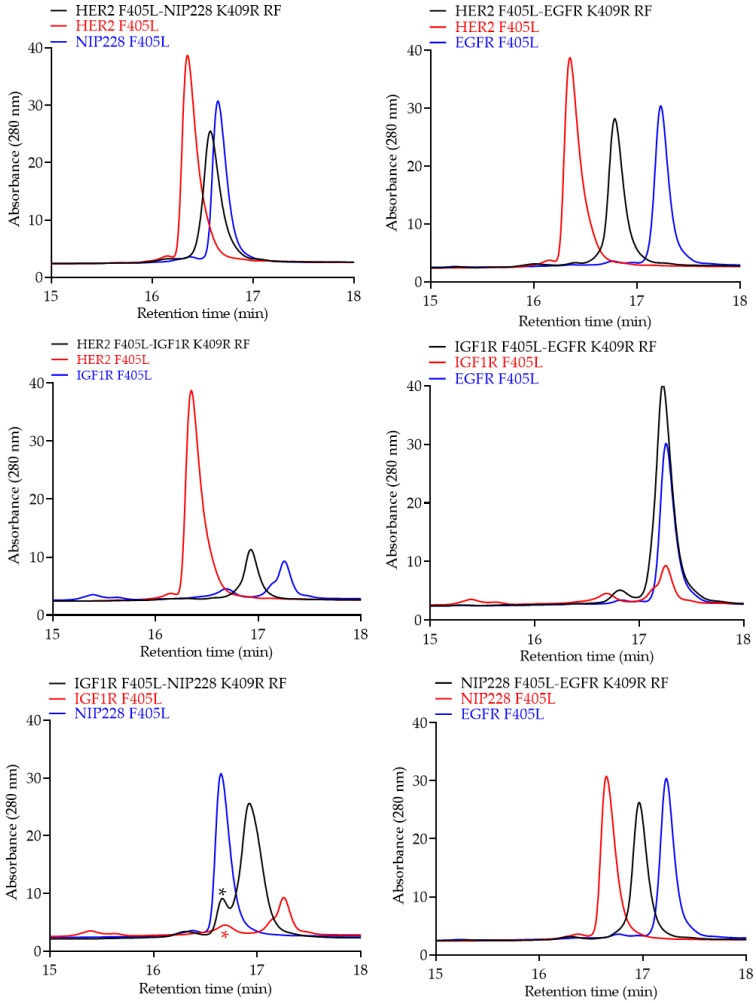
Intact reverse phase liquid chromatography (RPLC) profiles of purified monovalent bsAbs (black traces) compared to the two parental mAbs (red and blue trace). The red asterisk symbol may represent a non-reactive half antibody. This species may contribute to a slightly lower bsAb formation, as shown by the first peak of the bsAb (black asterisk symbol), which is a combination of the anti-IGF1R half antibody and the non-reacted NIP228 antibody.

Monovalent bsAbs were further analyzed via intact reverse phase liquid chromatography (RPLC). Intact RPLC confirmed the predicted retention time of the heterodimerized monovalent bsAbs species (black trace, Figure 6) as compared to the two parental antibodies (red and blue trace, Figure 6) for all six monovalent bsAbs.

**Figure 7:**
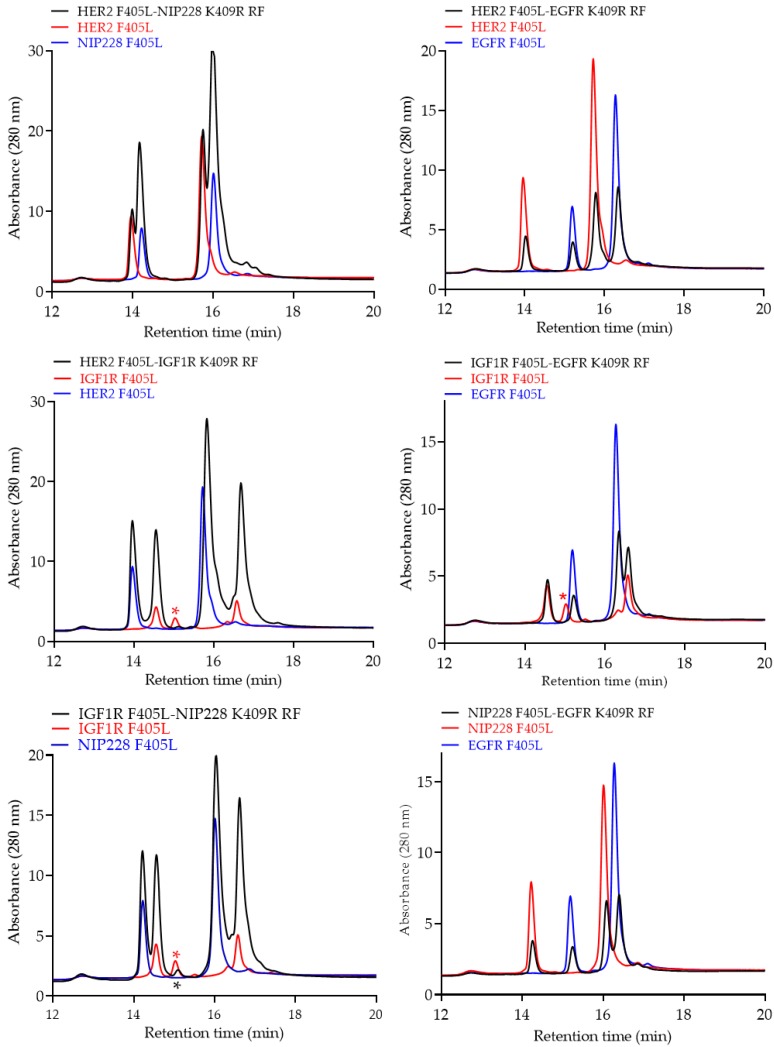
Reduced reverse phase liquid chromatography (RPLC) profiles of purified monovalent bsAbs (black traces) compared to the two parental mAbs (red and blue trace). The red asterisk symbol represents a molecular species, as described in Figure 6. As shown in RPLC (Figure 6), this species is carried over in the bsAb preparation (black asterisk symbol).

Furthermore, monovalent bsAbs were analyzed via reduced RPLC, in which antibodies were reduced using 50 mM DTT. Parental antibodies had two unique retention times: one each for the light and heavy chains (red and blue trace, Figure 7). Heterodimerized monovalent bsAbs had four unique retention times (black trace, Figure 7), which overlapped with the two unique light and heavy chain species from the respective parental antibodies, thereby confirming that the individual components of the heterodimerized bsAbs are present.

**Figure 8:**
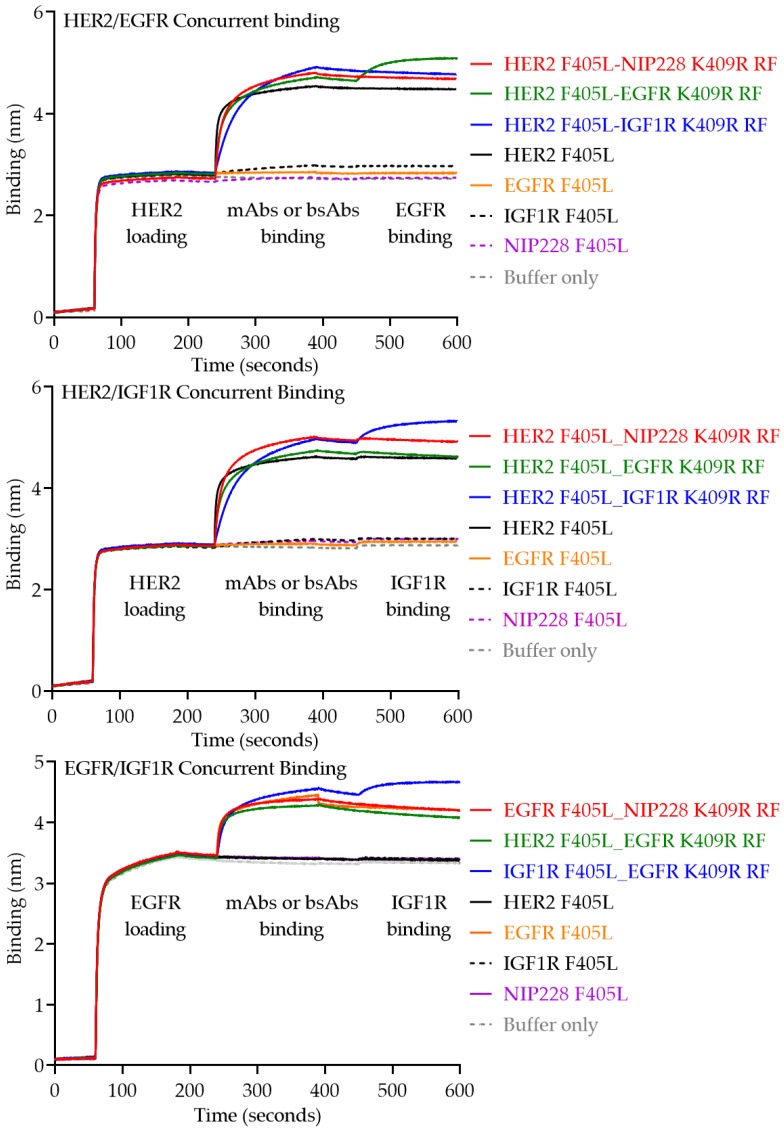
Biolayer light interferometry (BLI) traces for concurrent binding of monovalent bsAbs to two separate ligands. HER2-EGFR, Her2-IGF1R, and EGFR-IGF1R concurrent binding were analyzed in the top, middle, and bottom experiments, respectively.

Finally, to evaluate the capability for the monovalent bsAbs to simultaneously engage two distinct epitopes, concurrent binding assays were carried out via biolayer interferometry (BLI) with OCTET. The HER2, EGFR, and IGF1R ligands were utilized. Three different assays were utilized to individually evaluate HER2/EGFR, HER2/IGF1R, and EGFR/IGF1R concurrent binding. In the first assay (top panel, Figure 8), biotinylated HER2 was first loaded onto a streptavidin sensor, before then loading the parental antibodies as well as HER2-containing bsAbs, followed by the second ligand EGFR. In the second assay (middle panel, Figure 8), biotinylated HER2 was first loaded onto a streptavidin sensor, before then loading the parental antibodies as well as HER2-containing bsAbs, followed by the second ligand IGF1R. Finally, in the third assay (bottom panel, Figure 8), biotinylated EGFR was first loaded onto a streptavidin sensor, before then loading the parental antibodies as well as EGFR-containing bsAbs, followed by the second ligand IGF1R. As expected, in the first two assays (top and middle panels, Figure 8), parental and bsAbs containing a HER2 entity were able to bind to the HER2 ligand. However, only the bsAbs containing both HER2/EGFR (top panel, Figure 8) and HER2/IGF1R (middle panel, Figure 8) were able to then subsequently bind the final ligands, EGFR and IGF1R, respectively. Additionally, in the third assay (bottom panel, Figure 8) parental antibodies and bsAbs containing an EGFR entity were able to initially bind the EGFR ligand, but only the bsAb containing both EGFR/IGF1R was able to then bind the final ligand, IGF1R.

## 4. Discussion

Over 50 bsAbs are currently undergoing clinical trials and numerous efforts are being made to facilitate the generation and evaluation of these therapeutically promising molecules [23]. The utility of the platform described herein is that the purification process is highly efficient and yields high-quality monovalent bsAbs directly from cell culture supernatants, thus bypassing multiple purification and clean up steps (Figure 2). Unlike numerous other platforms, this platform ensures proper light chain pairing and effective heavy chain heterodimerization without the need for removing mispaired, unpaired, or placeholder “dummy” chains. Furthermore, due to the incorporation of the Protein A ablating H435R and Y436F mutations, there is more forgiveness for potential inaccurate protein quantitation if the K409R_RF entity saturates and drives the reaction to ensure all F405L entities are heterodimerized. As demonstrated by SEC, LCMS, and RPLC (Figure 4, Figure 5, Figure 6 and Figure 7), this platform results in highly monomeric monovalent bsAbs of expected molecular weights. Moreover, the monovalent bsAbs prepared with the methods describe herein are functional as demonstrated by their concurrent binding to antigens (Figure 8).

## 5. Conclusions

bsAb can simultaneously bind two epitopes on the same or on different antigen. Therefore, bsAb facilitate can novel modes of action, which cannot be achieved by conventional monospecific IgGs. One critical component of bsAb development is the identification of target pairs of antibodies with synergistic effect, necessitating the development of high-throughput approaches. To address this hurdle, we developed a platform to rapidly assemble and evaluate candidate bsAb for improved biological functionality in high-throughput format directly from culture media. We anticipate that our platform can expedited the development of bsAb therapeutics.
